# Grave-to-cradle: human embryonic lineage tracing from the postmortem body

**DOI:** 10.1038/s12276-022-00912-y

**Published:** 2023-01-04

**Authors:** Seock Hwan Choi, Eu Jeong Ku, Yujin Angelina Choi, Ji Won Oh

**Affiliations:** 1 Department of Urology, School of Medicine, Kyungpook National University, Daegu, Republic of Korea; 2 Bio-Medical Research Institute, Kyungpook National University Hospital, Daegu, Republic of Korea; 3 Department of Internal Medicine, Chungbuk National University Hospital and Chungbuk National University, College of Medicine, Cheongju, Republic of Korea; 4 Department of Anatomy, Yonsei University College of Medicine, Seoul, Republic of Korea; 5 Brain Korea 21 PLUS Project for Medical Science, Yonsei University College of Medicine, Seoul, Republic of Korea

**Keywords:** Cell lineage, Embryology, Cells

## Abstract

Curiosity concerning the process of human creation has been around for a long time. Relevant questions seemed to be resolved with the knowledge of how cells divide after fertilization obtained through in vitro fertilization experiments. However, we still do not know how human life is created at the cellular level. Recently, the value of cadavers as a resource from which to obtain “normal” cells and tissues has been established, and human research using postmortem bodies has attracted growing scientific attention. As the human genome can be analyzed at the level of nucleotides through whole-genome sequencing, individual cells in a postmortem body can be traced back to determine what developmental processes have transpired from fertilization. These retrospective lineage tracing studies have answered several unsolved questions on how humans are created. This review covers the methodologies utilized in lineage tracing research in a historical context and the conceptual basis for reconstructing the division history of cells in a retrospective manner using postzygotic somatic variants in postmortem tissue. We further highlight answers that postmortem research could potentially address and discuss issues that wait to be solved in the future.

## Development: Mapping the history of individual cells

Identifying all the precursors of an individual cell, called lineage tracing, is allowing researchers to gain a new level of understanding of human development. Cells naturally accumulate non-harmful mutations over their lifetime. Using whole-genome sequencing of individual cells, researchers can track accumulation of these mutations as cells divide and migrate, to make a cell-level map of development. Ji Won Oh at Yonsei University College of Medicine in Seoul, South Korea, and co-workers have reviewed recent developments in lineage tracing performed using individual cells from postmortem bodies. They report that lineage tracing provides insight into the asymmetric contributions made by early cells, and into which cell types build each organ. They hope the field will produce a map of cell divisions and cellular fate from fertilized egg to mature adult, deepening understanding of human development.

## Introduction

**Fig. 1:**
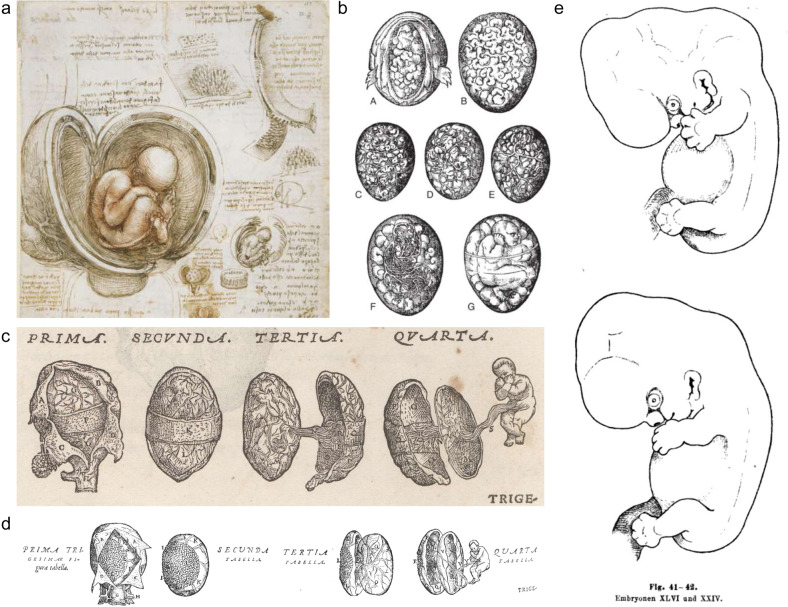
Old drawings of the human embryo. **a** Fetus in the womb from Leonardo da Vinci’s sketches and notes, 1510. **b** The fetal creation process from Jacob Rueff’s De Conceptu et Generatione Hominis, 1554. **c** The placenta and the fetus drawn from De humani corporis fabrica libri septem, original edition, 1543, and (**d**) second edition, 1555. **e** Drawing of the embryo from His in *Anatomie menschlicher Embryonen*, 1880.

How a person is created and how individual organs develop have long remained unanswered questions. Leonardo da Vinci left a sketch of how an embryo was formed in 1510 (Fig. 1a). Andreas Vesalius drew an intact fetus with umbilical cord and placenta in his book, De humani corporis fabrica libri septem (“On the Fabric of the Human Body in Seven Books”), in both the original (1543) and second editions (1555) (Fig. 1c, d). In 1554, based on the teachings of Aristotle, the fetus was believed to be the product of the coagulum formed by blood and semen, an idea that was passed down until the 18th century (Fig. 1b). Uncertainty and assumptions about how a person is created were clarified, to some extent, macroscopically through the work of Swiss embryologist Wilhelm His^1^ (Fig. 1e), the Carnegie Stage study in 1942 by Streeter^2^ and later in 1987 by O’Rahilly and Müller^3^. Therein, the stages of embryo development were divided, in detail, into 23 stages, covering fertilization to the gestational age of 10 weeks.

Advances in modern embryologic research have been bolstered by advances in in vitro fertilization (IVF) technology, which Robert Edward developed for the treatment of infertility^4^. IVF involves fertilization of human eggs with sperm outside the body and the artificial insertion of the embryo into a uterus to give birth to a human being. The inherent nature of IVF has made it possible for scientists to observe fertilized eggs externally. From this, detailed knowledge of developmental processes after fertilization, especially cleavage, and the processes of making morulae and blastocysts have been obtained. However, the idea that individuals can be created through these artificial processes, as well as the concept that IVF cells can become human beings, have raised ethical concerns. Accordingly, a 14-day rule, permitting the study of IVF embryos only up to 14 days after fertilization or until a primitive streak occurs, was established in 1979^5^. This 14-day rule is agreed upon between the scientific community and social ethics leaders and is enforced in all IRB regulations. Thus, human embryo research into developmental processes occurring later than 2 weeks after fertilization, including gastrulation, organogenesis, somite development, neurulation, and the development of limbs and genitalia, has had to seek other experimental directions.

In this paper, we intend to discuss lineage tracing and the process of conducting human embryology research using recently developed next-generation sequencing (NGS) technology, with a focus on postmortem human tissues. In particular, we cover the development of lineage tracing tools in embryology, including the use of somatic variants, in detail. Additionally, we address the biological and scientific background of how this development was possible, as well as challenges and hypotheses that need to be solved in the future.

### Lineage tracing using somatic variants

Lineage tracing is defined as the identification of all progenies of a single cell and how each individual cell divides and determines its fate^6^. In animal models, experimental interventions have made it possible to conduct sophisticated and meticulous lineage tracking with the support of many repetitive experiments. For example, Conklin’s work in 1905 showed how a multicellular organism (ascidian egg) develops through several cell divisions^7^. In particular, he investigated the development of early embryonic cells by tracking the intrinsic yellow crescent cytoplasm observed in muscle and mesenchymal lineage cells in ascidian eggs under specific optical conditions^8^. Developmental processes from a single fertilized egg to gastrulation were tracked, revealing time points for the differentiation of the endoderm, ectoderm, and mesoderm. He also proved the concept of “cell lineage” based on the study of annelids by Edmund Wilson, who suggested that specific cells have their individual “fates” before their division in succession^9^.

In 1983, a milestone study traced the entire process from fertilization to adulthood through lineage tracing. Sir John Sulston and his colleagues reported how the early development of *Caenorhabditis elegans* proceeded and when cells’ fates were determined^10,11^. They found that cells can spontaneously disappear, and upon confirming that these were not errors of the experimental observation, they arrived at the concept of programmed cell death for the first time.

Traditional studies of cancer have indicated that mutations in tumor suppressor genes and oncogenes can lead to cancer. Mutations in these genes are collectively called driver mutations. This concept of driver mutations accelerated the study of how normal cells change to tumor cells in cancer^12^. However, when various cancers were analyzed through NGS, researchers discovered, in addition to mutations in major cancer-related genes, mutations throughout the entire genome: in 1989, mutations were found to be present in various areas of the noncoding region of chromosome 17 in astrocytoma^13^. Although the function of these mutations has not been clearly elucidated thus far, these mutations have been called “passenger” mutations, in contrast to driver mutations^14^.

The fact that such mutations exist in cancer cells has enabled cancer biologists to conduct retrospective lineage tracing in patients with multiple cancers. Taking advantage of the simple fact that mutations are inherited by daughter cells, it seems possible to reconstruct the “life history” of cancer cells through mutations. The challenge of this was first reported in 2012^15^. To investigate intratumor heterogeneity, the authors utilized somatic variants in individual cancer cells, making a phylogenetic tree of cancers based on patterns of shared and private mutations with temporal dynamics of the somatic mutations. The tree visualized how and when metastases evolved at the cellular level and traced them back to the progression from normal tissue to cancer. As this methodology requires only dissected cancer tissues from patients without any experimental intervention, retrospective lineage tracing of human cells is possible at the in vivo level without ethical concerns. Similar methodologies have been applied to reconstruct the history of human cancer cells using somatic variants underlying cancer cell heterogeneity^16^ in many different cancers, including leukemia^17^, glioblastoma^18^, breast cancer^19,20^, myeloproliferative neoplasms^21^, muscle-invasive bladder cancer^22^, kidney tumor^23^, and adenoma^24^.

In general, the frequency of somatic mutations in cancer cells increases because of driver mutations^25–27^. However, studies of cancer and other sporadic diseases have revealed that many cancer-free controls exhibit somatic mosaicism, even under normal conditions^28^. Eventually, researchers discovered postzygotic variants (PZVs) in early embryonic stages. For example, long interspersed nuclear element-1 (LINE-1) retrotransposons can be altered in terms of copy number during embryogenesis^29^, resulting in mosaicism of neuronal progenitor cells and adult brain tissue^30^. Structural variations, such as copy number variations, have also been reported in normal tissues^31^. Additionally, during cleavage of the human embryo, chromosomal instability is relatively common, both pathologically and physiologically^32,33^.

Mosaicism is a state in which different genotypes (DNA) exist within an individual^34^. It is mainly known to be involved in pathological diseases of the human body. In the case of skin mosaicism, due to a significant phenotype compared to other normal skin tissues, it becomes much more visible. Indeed, somatic mosaicism, or PZVs, can occur throughout the entirety of human life^35^. Research has confirmed that the somatic mutations that create these mosaics are randomly distributed across the entire human genome^36^. The average base-substitution somatic mutation rate in normal tissues is estimated at 3.05 mutations per cell (diploid genome) per cell division (pcpcd) in retinal development, 0.88 mutations pcpcd in intestinal epithelial cells, 4.54 mutations pcpcd in B and T lymphocytes, and 1.05 mutations pcpcd in fibroblasts^37^. Our previous study reported 3.8 (range, 1.4 to 6.3) mutations pcpcd in the earliest cell division up to four cell stages, which is similar to other reports, but 1.2 (range, 0.8 to 1.9) mutations pcpcd after four cell stages in early human embryogenesis^38,39^. In normal tissue, the estimated rate of mutation accumulation is 33 per year per diploid genome in the liver;^40^ 36 per year per diploid genome in adult stem cells of the colon, small intestine, and liver;^41^ 14.2 per year per diploid genome in hematopoietic stem cells and multipotent progenitor cells in bone marrow;^42^ and 43.6 per year per diploid genome in colorectal epithelial cells^43^.

**Fig. 2:**
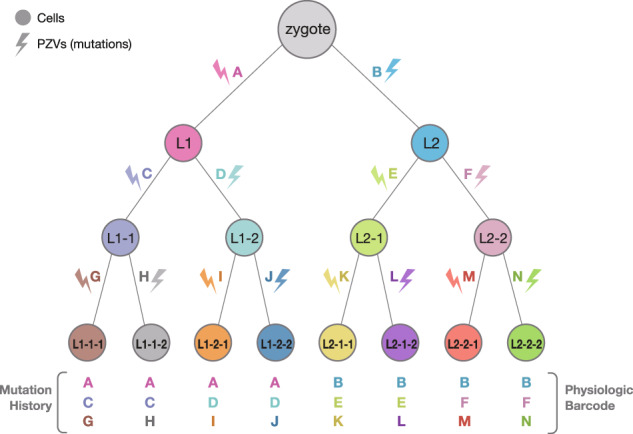
A theoretical example of sequential mutation events of the first three cell divisions. When the zygote divides into two progenies, each progeny having a single different mutation, A and B, these mutations of lineages 1 (L1) and 2 (L2) can be distinguished. After two more divisions, each cell has different combinations of sequential mutation histories. Based on shared and private individual mutations, the phylogenetic tree of each cell can be reconstructed.

Theoretically, if a single mutation accumulates at each cell division, as cell division continues and other mutations accumulate during each division, all of the cells of an individual can have different DNA (Fig. 2). Since these cells divide thousands of times over a lifetime, it can be inferred that the mutations accumulated during each division ultimately result in each cell having its own unique DNA pattern. In the case of PZVs occurring in early embryogenesis, since a large number of progeny cells contain those mutations, it is possible to completely reconstruct the initial developmental processes of these cells depending on whether they have shared mutations or not. That is, with each division throughout the life of an individual, genetic mutations accumulate, suggesting that the history of cell division can be tracked^44^. This concept was first validated in mice using clones obtained from the organoid technique^45^. Eventually, PZVs were found to enable retrospective lineage tracing in the study of human embryogenesis^38,46–51^.

### Postmortem tissue for advanced research

In most animals, including humans, a fertilized egg keeps dividing and eventually differentiates into the ectoderm, mesoderm, and endoderm during development. Thus, to correctly reconstruct a cell’s lifetime, cells from these three germ layers must be obtained within an individual to reconstruct their processes. For example, ectoderm-derived tissue, such as corneal epithelial cells and skin epithelium; mesoderm-derived tissue, such as blood cells and fibroblasts; and endoderm-derived tissue, such as colon crypt cells and hepatocytes, should be simultaneously obtained from one individual for the proper reconstruction of embryogenesis. This experimental strategy can determine the timing of divergence from each germ layer. However, for samples from a surgical patient obtained in general medical research with informed consent, it is quite challenging to obtain cells of the above three germ layers at the same time. On the other hand, in the case of a donated postmortem body, it is possible to secure enough cells derived from these three germ layers in one individual.

Most human cadavers in the past were utilized for anatomy education, understanding structure, and solving superficial pathologies, while in modern times, advanced biological methodologies have been applied to better understand the in vivo state of the human body^52,53^. Efforts such as the Genotype-Tissue Expression (GTEx) project, initiated by the National Institutes of Health to collect various postmortem normal tissues that reflect the in vivo state and cannot be obtained from patients, have increased our understanding of molecular biological aspects, specifically at the transcriptome level^54^. The GTEx project has gathered 17,382 samples across 54 nondiseased tissues from 948 individuals, including data from gene expression profiling, whole-genome sequencing (WGS), and RNA-seq^55^. They also evaluated the usefulness of postmortem intervals at the level of the transcriptome. Data from 1 h to 27 h after death are reported in detail concerning how tissues change over time at the molecular level and how transcriptomic signals are changed^56^. According to studies of gene expression profiles in postmortem tissue, the postmortem interval (PMI) of RNA degradation differs between tissues, genotypes, and specific genes; for example, the cerebellum and pituitary gland show relative stability in terms of PMI-associated genes, while esophageal mucosa and whole blood exhibit many altered expression patterns of PMI-associated genes.

Transcriptome data fluctuate with postmortem RNA degradation, which requires strict control over the “normal” structure, but DNA in postmortem tissues is preserved safely. However, in lineage tracing of embryogenesis, it is necessary to secure a sufficient amount of intracellular DNA originating from a single cell or to cultivate a clonal cell from postmortem tissues in vitro. Since error rates for nonreference genotype cells range from 0.1% to 0.6%, depending on the depth of coverage or the platform^57,58^, finding a somatic variant that occurs less frequently than the error rate of NGS requires the use of specialized experimental strategies.

## Methods for the detection of early embryonic mutations (EEMs) for retrospective lineage tracing of postmortem tissues

Two main challenges hinder embryonic lineage tracing using the postmortem body. First, the genome of single cells must be obtained from a variety of different anatomical areas that accurately reflect the developmental processes in the three germ layers. Second, because the incidence rate of errors in NGS exceeds the incidence rate of PZV events in vivo, which is from 0.1 to 3 mutations per cell per cell division^39^, it is very important to set up an experimental strategy to pinpoint the somatic variants accurately in wet experimental designs and bioinformatical analysis. Currently, to proceed with retrospective lineage tracing of postmortem tissues, somatic variants are explored in three different ways.

### Clonal expansion in vitro from various anatomical locations and organs

**Fig. 3:**
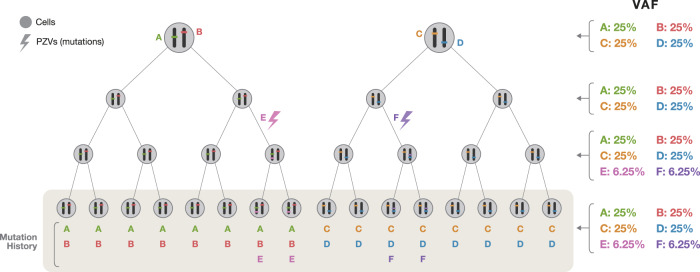
A theoretical example of expansion from two different lineage cells. When cells in different lineages divide equally, the variant allele frequency is much lower than 50%. In this example, 25% of all genuine mutations are represented, except for culture-induced mutations (E and F), which are lower than 25%. In this case, it is relatively difficult to determine in which cell the mutation originated. If the origin of cells is polyclonal in LCM, the situation is much more complicated.

Since clonal expansion originates from a single cell, “expansion as a replication tool” retains all mutations that reflect the history of a cell (Fig. 3). Clonal expansion for retrospective lineage tracing requires not only well-trained developmental biologists who have expertise in culture but also anatomists who can dissect and quickly preserve proper organs. Precise primary culture methods and advanced clonal expansion culture techniques are also essential. At the same time, to obtain a sufficient level of DNA from a clone, it is necessary to culture cells for >2 months. These clones produced by clonal expansion methods can provide relatively pure signals of PZVs with minimal artifacts in NGS. To date, protocols have been established for obtaining clonal lines of mesodermal origin, such as skin fibroblasts^38,49^ and muscle myocytes from head to extremities^38^, hair dermal papilla stem cells^59^, and hematopoietic stem cells;^60,61^ endodermal origin, such as organoids of the stomach^45^, liver^62^, small intestine, colon^41^, and prostate;^63^ and ectodermal origin, such as cornea keratinocytes^38^ and neuronal progenitor cells^46^ of the fetus. Although it is very challenging and labor-intensive to obtain large numbers of clones from an individual, especially a cadaver, it is believed that a clone can provide the purest somatic mutation signals of the entire history of cellular division for WGS, compared to other methods, such as laser capture microdissection (LCM) and single-cell DNA sequencing.

Recently, an attempt has been made to use induced pluripotent stem cells (iPSCs) to obtain clonal cultures of fibroblasts^49^. The researchers used transient episomal vectors expressing human Oct4, Sox2, Lin-28, Klf4, and L-myc to culture the iPSCs of fibroblasts^49^. Although iPSC clones were created using fibroblasts for the proof of concept for the detection of early PZVs, iPSC techniques could be utilized for cells with limited proliferative potential. Based on our experience, it can be difficult to obtain clones from ectodermal origins with a sufficient amount of DNA for WGS. Though the success rate of primary culture per location of the epithelium is 43%–62%, which is similar to that of fibroblasts, the success rate of clonal expansion per location of the epithelium is relatively very low at approximately 5%–8%, while that of fibroblasts is 30%–45%. These empirical results indicate the need to establish different strategies for the epithelium, where there is a low potential of establishing clones. In this respect, iPSC techniques may play a pivotal role in minimized artifact DNA replication machinery for low-potential proliferative cells, such as epithelial cells or differentiated cells, not for pluripotency purposes.

Clonal expansion in vitro has some disadvantages: it takes tremendous initial effort, and only cells that can grow for a long time are obtained due to selection bias. In addition, it is a prospective experiment that must be carried out within 36 hours after death. However, once obtained, all the mutations a cell has developed in its lifetime can be investigated, including those in mitochondrial DNA. Clonal expansion may generate a few (<10) culture-induced mutations that are shared by most of the cultured cells from individual clones. We have found that the vast majority of mutations identified in clones are genuine^38^.

### Laser capture microdissection (LCM) from various anatomical locations and organs

The second method is LCM^64,65^. LCM is commonly used in retrospective lineage tracing in the liver^40,66^, colorectal epithelial cells^43^, colon^67^, endometrial epithelium^68^, placenta^51,69^, diverse glands^48^, and normal tissue mutational profiles^70^. The scientific rationale is that certain stem cell populations grow clonally within a specific niche in vivo^71–73^. LCM can physically separate these natural cellular clusters of dominant clones and provide solid information on somatic mutations among WGS clones.

Unlike clonal expansion, this method has some advantages: it can be performed histologically, it requires less labor, and it can be used for various in vivo clonal expansion tissues. Additionally, LCM methods have the advantages of preserved spatial distribution and histopathological features of cells within a tissue^65^. At the same time, since the LCM experiment can be carried out after storage of the tissue, it has the strength of being able to be performed electively at the desired time.

However, LCM cannot be applied to several types of tissues or cells, such as fibroblasts and brain and heart tissue, that do not naturally create clone structures in vivo. Although an apparent clone structure may be seen on histology, this could be the result of contamination by other cells, resulting in polyclonal areas that waste money or require sophisticated bioinformatical analysis (Fig. 3). In addition, the quality of DNA could be relatively low and bioinformatically difficult to analyze due to signal noise. Nevertheless, LCM methods are the leading methods for the study of embryogenesis using somatic variants and are expected to be developed for almost every internal organ and endodermal lineage for higher resolution of retrospective lineage tracing maps.

### Single-cell DNA WGS using whole-genome amplification (WGA) with or without high-depth bulk sequencing (>200X)

A third method is WGA of a single cell^47,74^. This method has the advantage of being able to acquire a large number of cells simultaneously and is relatively easy to perform without selection bias. At the same time, it provides flexibility for a diverse range of cells, including nondividing cells, such as neurons. However, it is difficult to distinguish between genuine PZVs and de novo mutations occurring in the early stage of the amplification process^75^. At the same time, “allele dropout” and intrinsic errors during amplification are relatively common^76^, and therefore it is not preferred as a single method for detecting PZVs.

To overcome these limitations, a recent study combined high-depth bulk sequencing data to detect shared PZVs and then confirmed the results via single-cell target sequencing for the reconstruction of early embryogenesis^50,77^. Using the fact that early EEMs are shared in many cells of the body, resulting in a high variant allele frequency (VAF) in bulk tissue in WGS, bulk sequencing is performed at a depth of 200X or more to find EEMs with statistical confidence^78,79^. Then, the exact locus positions of the found EEMs are validated in a large number of cortical single cells, leading to the precise embryonic lineages of each of these cells.

This bulk sequencing approach cannot lead to the discovery of PZVs as physiological barcodes of the entire lifespan. However, anatomically refined high-resolution bulk sequencing of more than two samples from one individual can complement clonal strategies and provide information on early lineage^79^. One study sought to determine shared PZVs for the reconstruction of early lineages, which are relatively frequent^80^. Indeed, WGS at a depth of approximately 100X can detect somatic variants during early embryogenesis, which have different mutational profiles, such as replication timing and chromatin status of the later tissue-specific clonal expansion^81^.

Several single-cell WGA methods have been developed thus far^76^, and various computational approaches have been applied to identify somatic variants^82^. Among them, the recently developed isothermal WGA method, primary template-directed amplification (PTA), is attracting attention^83^. Since PTA is relatively more uniform and accurate than the existing single-cell WGA methods, such as multiple displacement amplification (MDA), the accuracy and sensitivity of somatic variant calling are improved^83,84^. As a result, its use is increasing in neuron and brain research, where there is no histological niche to perform LCM or in vitro clonal expansion^78,85^. PTA is expected to be applied in other organs in various experimental strategies, along with bulk WGS sequencing.

### Findings of Human Embryogenesis from Postmortem Lineage Tracing

Proof-of-concept studies for retrospective lineage tracing, which used PZVs as physiological barcodes to reflect cellular lineage history, have provided potential answers to unsolved questions in human embryology. Recent studies have presented evidence for various human embryologic hypotheses concerning when the fates of the trophectoderm and inner cell mass (ICM) are determined, embryologic asymmetry in the human body, and in what order each cell determines their fate in terms of the three germ layers, anatomical right-left commitment, and organogenesis.

Based on our data with mathematical simulations, the time point for the specification between the trophectoderm and ICM occurs following the third or fourth division of a zygote: five of eight cells at the third division (or 10 of 16 cells at the fourth division) are considered to be of the trophectoderm lineage, which forms the placenta, while the remaining three cells contribute to the fetus proper^38^. These three cells eventually develop into the ICM, differentiating into every organ of the human body. Because of an odd number of founder cells in the fetus, the cellular contribution to the whole body is considered to be asymmetric (approximately 1:2 at the first division of the zygote). Based on seven postmortem bodies in our study^38^ and other related embryogenesis studies, including five fetal postmortem bodies^46,69^, six postmortem bodies^48,50^, and three living individuals^49,61^, we can statistically generalize the scientific evidence of this asymmetric contribution of the founder cells to the human body according to the patterns of shared and unique PZVs in each clone within an individual.

Based on the analysis of PZVs of clones within an individual, the shared patterns of PZVs among clones as a marker for early embryonic lineage could be utilized for further analysis. For example, when mutually exclusive PZVs are identified, they could be used to identify the specific lineages of clones carrying these PZVs. Because the specific genomic position of PZVs can be defined for specific lineages after the analysis of clones (capture phase), targeted deep sequencing can be conducted on the genomic position of PZVs for target organs to determine the contribution of that lineage to a target organ (recapture phase). This approach has helped determine that the contribution of first lineage cells of zygotes to the body as well as a specific organ is not identical but asymmetrical, suggesting that a cellular bottleneck contributes to organogenesis. Indeed, the degree of asymmetric contribution of the first two reconstructed founder cells to the entire body appears to be highly variable among individuals^38,48–50^. This may be due to the existence of a strong bottleneck when the embryonic blastomere is committed at the blastocyst stage or presumptive apoptosis of early founder cells during embryogenesis.

**Fig. 4:**
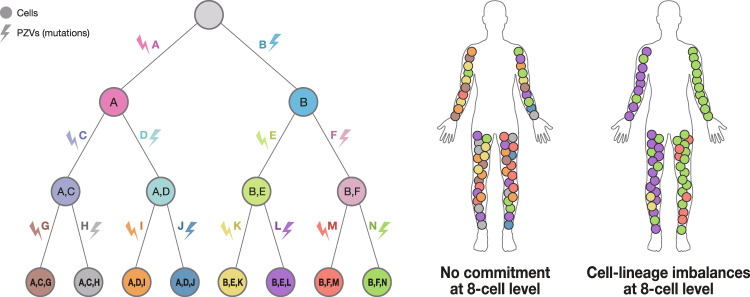
A theoretical example of cell-lineage imbalance. If a specific cell contributes more when making a certain tissue or organ, the proportion of that cell in the tissue will be relatively larger than that of other cells. Conversely, if every cell contributes equally to making a particular tissue, it can be inferred that the cell’s fate has not yet been decided. In this case, since the fate is determined as division progresses, the division stage of the cell is also important. This principle makes it possible to infer when each cell’s fate has been decided.

Meanwhile, the commitment time of the three germ layers has been determined by focusing on the ectoderm^38^, primordial germ cells^48^ (PGCs), and cortical neurons^50^. After the analysis of several clones from the same germ layer or lineages, targeted deep sequencing facilitates the analysis of specific accumulation patterns of PZVs to represent lineages from this germ layer. In other words, if we conduct targeted deep sequencing of genomic positions for organs and bulk tissues, we can reasonably infer the contribution of certain lineages for that organ. This principle can be applied to the organs of each of the three germ layers or specific organs with early PZVs. The hypothesis is that if certain lineages are committed to an organ or germ layer, the proportion of PZVs exclusive to certain lineages would be much higher than that of PZVs of other lineages, resulting in genetic imbalance (Fig. 4). Applying the principle of the genetic imbalance of certain lineages to our previous five postmortem datasets showed that ectodermal specification fates were decided earlier than those of other germ layers^38^. However, the time points of the endoderm and mesoderm have not yet been determined because the number of clones investigated was too small to pinpoint the fate decision stage. Future higher resolution studies with more clones are expected to facilitate the discovery of the exact timing of germ layer commitment and organogenesis.

A human blastocyst is composed of two groups of blastomeres, the ICM and the trophectoderm^86^. The ICM (embryoblast) differentiates into two distinct structures: the primitive ectoderm (epiblast) that forms the amnion and the three germ layers and the primitive endoderm (hypoblast in humans) that forms the visceral yolk sac^87^. The trophectoderm becomes the syncytiotrophoblast during its development into gastrulation. It is well known that the visceral endoderm, with extraembryonic lineage, is intercalated into the hypoblast in mice^88,89^, and genetic labeling methods have revealed that a small portion of the endoderm is composed of those lineages and a large portion of the endoderm is from the epiblast. However, in humans, little is known about the involvement of extraembryonic lineages in the human body proper. Based on LCM of seminiferous tubules in male postmortem bodies, PGCs have been found to share relatively low numbers of PZVs with other tissues, suggesting that they might have different developmental trajectories^48^. Some studies have shown that there are no commonly shared PZVs between PGCs and matched tissues. This may suggest that there is a small number of cellular bottlenecks that produce PGCs and a relatively early divergence of PGCs from other blastomeres. At the same time, the gut epithelium shares mutations with the placental villi mesenchyme, which is of extraembryonic lineage^69^.

Applying the concept of cell-lineage imbalance, the contribution of specific lineages for the right/left tissues (mainly the four extremities) has also been reported^38^. Right/left emergence and imbalance of first lineages appeared to be acquired at the 1^st^–4^th^ estimated cell generation before gastrulation in five postmortem human bodies. The right/left kidneys, lungs, and lobes of the liver, in particular, show consistent right/left imbalances, indicating that right/left commitment occurs before organogenesis. However, the stomach shows a relatively later imbalance at the 9^th^-17^th^ cell generation, indicating that the stomach is made of the center of the embryo.

**Fig. 5:**
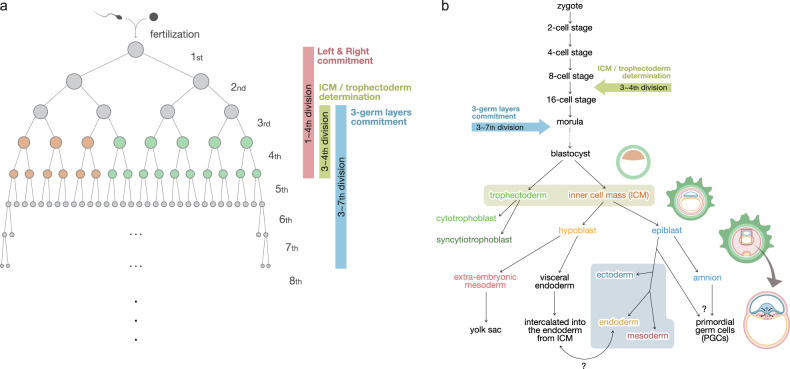
Schematic representation and overview of early human embryogenesis. **a** Schematic representation of the specific timing of early human embryogenesis based on clonal imbalance. **b** Schematic temporal overview of different lineage commitments in early human embryogenesis.

Based on data from the postmortem body thus far, it can be concluded that the following temporal sequence takes place during human embryogenesis (Fig. 5): After fertilization, the fates of the ICM and trophectoderm are determined approximately in the 3^rd^ or 4^th^ cell generation. It is estimated that three of eight cells resulting from the third division form the ICM, and five form the trophectoderm. Left/right commitment seems to appear at the 1^st^ to 4^th^ cell generation. After that, the ICM separates into epiblasts and hypoblasts. Hypoblasts divide into the visceral endoderm and extraembryonic mesoderm. The visceral endoderm later undergoes intercalation with the endoderm from the ICM, and the extraembryonic mesoderm becomes primitive blood, taking part in yolk sac hematopoiesis. The epiblast is divided into three germ layers and an amnion, which will later become a partial source of PGCs. Among the three germ layers, the fate of the ectoderm is determined first, after which the mesoderm and endoderm seem to separate. This process is expected to occur approximately in the 3^rd^–7^th^ cell generation, after which organogenesis begins and regional imbalance occurs. The composition of the early embryonic lineages seems to be changed by external stimuli and intrinsic properties, leading to cellular stochasticity.

Retrospective embryological research using postmortem bodies has been conducted on approximately 25 people. Intrinsic mutation rates are very important in studying and precisely reconstructing phylogenetic embryologic lineages. In other words, a key point to ensuring that an experimental plan is successful is that mutations must occur at a sufficiently high frequency. Our experience and other studies have indicated that mutations in early human development occur at an average of 0.1 to 3 mutations pcpcd. However, some individuals have many early mutations, while others have very few. Theoretically, in instances of mutation rates less than 1 pcpcd, the full reconstruction of embryogenesis would be very challenging due to the lack of PZVs to mark the cellular lineages, resulting in polytomy, a single node with more than two descendent branches, not dichotomy, a node with only two descendent branches. Indeed, many cases exhibit multifurcation, not bifurcation, in the early stages due to the low mutation rate, leading to uninformative division. Although instances of lower mutation rates can be compensated for by solid statistical methods, future studies of a more precise estimate of specific commitment at the level of cellular generation require special experimental strategies.

### Future directions and unresolved questions

The cellular lineage field of human developmental biology is still in its infancy. Although the commitment trajectory of 40–277 clones with precise phylogenetic lineage trees was determined from various individuals, the number of cells was insufficient to reconstruct the commitment fate of human blastomeres to the entire human body. In addition, other studies have revealed the developmental processes of humans by partially focusing on specific anatomical organs either from living individuals or patients, such as the liver^40,66^, esophagus^90^, endometrium^68^, blood^60^, and brain^50^, with similar experimental strategies, although they explain the process only partially. The more we understand about early human embryogenesis, the more apparent the necessity of a higher resolution reference trajectory map becomes.

Although many questions have been answered, there are still many problems to be solved. First, it is necessary to pinpoint the exact time of commitment of each lineage in different developmental stages. This kind of work will present a clear map of the time when stochasticity is eliminated, resulting in commitment to a specific destiny. For example, a fibroblast is a unique mesodermal cell that originates from the nascent mesoderm at gastrulation. Since fibroblasts are transferrable during developmental stages and are located throughout the body in an adult, it is still inconclusive whether fibroblasts adjacent to other lineages are deterministic at the developmental stage or stochastic depending on the anatomical locations.

Second, as the behavior of “normal cells” changes with age, the exact evolutionary processes of cell division and the intrinsic differences in mutational rate in different cell lineages need to be investigated. Specifically, studies on which normal cells become cancerous and, conversely, which cells are more resistant to various mutations and maintain a normal state need to be conducted. In addition, it is necessary to study whether mutations that initially appear to have no function actually play a role. With age, some cells accumulate more mutations, and others carry relatively few mutations. It is crucial to ascertain whether these differences originate from a stage of stochastic development or are a property of chance acquired during development.

Third, questions concerning organogenesis should be addressed in more detail. Organogenesis is the phase of development that starts at the end of gastrulation after the commitment of the three germ layers. If sufficient clones are isolated from a specifically targeted organ, combined with other blastomere lineages from the same individual, researchers can determine when the most recent common ancestor (MRCA) commits to an organ and the minimum cell numbers of the MRCA at that stage. As the minimum pluripotent cell number is critical to mimic a physiological artificial human organ in vitro with a scaffold in regenerative medicine, this can provide insight into human embryology and practical evidence for the newly emerging field of mini organs.

Finally, a study of approximately 25 people thus far has yielded some generalizable knowledge, but it may still be an insufficient number to generalize to the human species. There is a need to study a much larger number of postmortem bodies to produce statistically significant data. In addition, to conclude the fate of each lineage commitment and to explore the unique developmental processes of the human species, it seems necessary to secure and analyze a larger number of clones of the various anatomical tissues from an individual, who contains more than 30 trillion cells.

## Conclusion

In this review, we summarized retrospective lineage tracing methods applied to the whole genome using postmortem human bodies. A massive number of experimental and bioinformatical methods are being developed for this field, necessitating understanding of the advantages and disadvantages of different experimental approaches, such as in vitro clonal expansion, LCM, and single-cell WGA with high-depth bulk sequencing for the detection of EEMs. In the future, utilization of a combination of two or more methods should be attempted for a better understanding of human embryology. Different groups use different terms with the same or similar meaning in this field, for example, somatic mutations, somatic variants, mosaic mutations (MMs), early mosaic mutations (EMMs), postzygotic variants (PZVs), and embryonic variants. Although there are subtle differences in the meaning of the terms, efforts to unify the terminology for this field will be necessary.

Postmortem body research, which involves obtaining different intact human organs simultaneously, is becoming a new means by which to study human embryology along with various NGS methods. It is paradoxical to be able to trace the birth process, which is the starting point of life, through the irreversible cessation of life in death. We are deeply indebted to those who donated their bodies, cells, and tissues for the advancement of this field.
